# Individualized mouse brain network models produce asymmetric patterns of functional connectivity after simulated traumatic injury

**DOI:** 10.1162/netn_a_00431

**Published:** 2025-03-20

**Authors:** Adam C. Rayfield, Taotao Wu, Jared A. Rifkin, David F. Meaney

**Affiliations:** University of Pennsylvania Departments of Bioengineering and Neurosurgery; University of Georgia School of Chemical, Material, and Biomedical Engineering; University of Virginia Department of Mechanical and Aerospace Engineering

**Keywords:** Computational model, Structural network, Functional network, Kuramoto model, Mouse

## Abstract

The functional and cognitive effects of traumatic brain injury (TBI) are poorly understood, as even mild injuries (concussion) can lead to long-lasting, untreatable symptoms. Simplified brain dynamics models may help researchers better understand the relationship between brain injury patterns and functional outcomes. Properly developed, these computational models provide an approach to investigate the effects of both computational and in vivo injury on simulated dynamics and cognitive function, respectively, for model organisms. In this study, we apply the Kuramoto model and an existing mesoscale mouse brain structural network to develop a simplified computational model of mouse brain dynamics. We explore how to optimize our initial model to predict existing mouse brain functional connectivity collected from mice under various anesthetic protocols. Finally, to determine how strongly the changes in our optimized models’ dynamics can predict the extent of a brain injury, we investigate how our simulations respond to varying levels of structural network damage. Results predict a mixture of hypo- and hyperconnectivity after experimental TBI, similar to results in TBI survivors, and also suggest a compensatory remodeling of connections that may have an impact on functional outcomes after TBI.

## Author Summary

Recent research has investigated the consequences of traumatic brain injuries by combining computational models of human brain activity and structural models of the whole human brain or cortex. As experimental injury research can only be conducted using animal models, we apply a simplified computational model of whole-brain dynamics, the Kuramoto model, to a mouse brain structural network. We tune our model to best predict measurements of functional connectivity recorded from 58 fMRI scans of mice and lesion the network model to explore the effects of injury. Our findings predict that functional connectivity may increase or decrease in various regions of the brain, even at a high injury level, which may aid in future predictions of cognitive impairments after brain injury.

## INTRODUCTION

Traumatic brain injury (TBI) is a pervasive health issue both globally and in the United States. The Centers for Disease Control and Prevention reported over 220,000 hospitalizations and over 60,000 deaths involving TBI in 2017 (Centers for Disease Control and Prevention, 2021). At least 75% of TBI cases are typically classified as concussions, also called mild TBI (Bazarian et al., 2005; Bruns & Hauser, 2003), and even these injuries can have high morbidity: Persistent postconcussion symptoms (PPCSs) can occur months after the injury for as many as 10%–20% of patients (Alves et al., 1993; Gozt et al., 2021; Signoretti et al., 2010; Yuh et al., 2013); can disrupt physical, cognitive, emotional, and sleep-related function; and may be measurable even 1 year postinjury (McMahon et al., 2014; Schneider et al., 2022). Despite a large literature describing the negative sequelae after even mild TBI, there is currently no method to clearly link the physics of the impact to the long-term patient outcome.

For over a decade, there has been increasing focus on describing the brain as a network system as a first step toward understanding how structural brain damage leads to cognitive impairment. In humans, the structural connectome (SC) is frequently estimated using information from diffusion-weighted MRI, while functional connectivity (FC) is often approximated using blood-oxygen-level-dependent functional magnetic resonance imaging (BOLD fMRI), which indirectly estimates brain activity through differences in local blood oxygenation (Sporns, 2013; van der Horn et al., 2017a, 2017b). Past work has shown that there are changes after TBI to both the SC (Klimova et al., 2019; Messé et al., 2011; Muller et al., 2021; van der Horn et al., 2017a; Wada et al., 2012; Ware et al., 2023) and FC (Hillary et al., 2014; Iyer et al., 2019; Lancaster et al., 2019; Li et al., 2020; van der Horn et al., 2017a). In some studies, changes in SC (Klimova et al., 2019; van der Horn et al., 2017b; Ware et al., 2020, 2023) and FC (Bittencourt et al., 2022; Iyer et al., 2019; Rangaprakash et al., 2017; van der Horn et al., 2017) are correlated with functional impairment in TBI patients. FC hyperconnectivity after TBI has been interpreted as a compensatory response, but its correlation with impairments may vary between studies. For example, frontal-temporal default mode network hyperconnectivity has been correlated with emotion recognition impairment during chronic moderate TBI (Lancaster et al., 2019), while frontal default mode network hyperconnectivity has been negatively correlated with depression scores during the subacute period following a single concussion (Zhou et al., 2012). In comparison, posterior default mode network hyperconnectivity has been positively correlated with PPCS patients’ depression scores (van der Horn et al., 2017b) and cognitive flexibility (Zhou et al., 2012). Given the nature of these clinical studies in human subjects where each injury is different from another, it is not straightforward to determine a relationship between brain damage and functional impairments.

Computational modeling techniques can aid in the study of both healthy and pathological brain states by providing theoretical models of functional network changes. Computational researchers use models of varying complexity, including representations of neural synchronization such as the Kuramoto model of coupled oscillators (Kuramoto, 1984), to explore how the brain’s physical structure can lead to the dynamic coupling of neural activity across brain areas. While the Kuramoto model is greatly simplified, its output can be compared with FC or dynamics obtained from resting-state BOLD fMRI (Cabral et al., 2011; Cocchi et al., 2016; Lee & Frangou, 2017) and used to make predictions about dynamical changes after perturbation (Gollo et al., 2017; Schmidt et al., 2015), structural lesions (Váša et al., 2015), and injury (Rifkin et al., 2022; Wu et al., 2022, 2022). Past work using connectomes modified to mimic the connectivity changes in TBI patients presented dynamics that differ from controls (Hellyer et al., 2015). Similarly, network changes produced by computational models of biomechanical injury have been used to separate impacts by injury outcome (Wu et al., 2022) and explore concussion vulnerability (Wu et al., 2022).

Despite these significant efforts to relate the human brain connectome to its functional neurodynamic state, these studies do not offer an ability to directly manipulate the physical connectome and study the corresponding effect on cognition. In comparison, preclinical models have an ability to precisely create consistent injury patterns across animals and are central to understanding TBI pathobiology, its relationship to cognitive deficits, and both biomarkers and possible treatments for TBI. The C57BL/6 mouse model is perhaps best suited for studying the relationship between lesions and cognitive behavior, as they are widely used in neuroscience, their cognition can be interrogated with a variety of behavioral experiments (Lueptow, 2017; Wheeler et al., 2013; Zhao et al., 2012), and their brain structure and function can be investigated using MRI techniques (Calabrese et al., 2015; Jonckers et al., 2015). Moreover, the Allen Brain Atlas project (Lein et al., 2007) provides a precise map of directional synaptic connections between different brain regions (Oh et al., 2014). To this end, recent studies have used these mouse connectome data to develop a Virtual Brain platform for simulating dynamics on the mouse brain (Melozzi et al., 2017), creating a reduced neural model of resting-state mouse brain fMRI (Melozzi et al., 2019). However, despite the potential impact of directionality on simulations of neural dynamics (Crofts et al., 2022), relatively few studies have explored the utility of the Kuramoto model on directed mouse brain networks developed from these data (Choi & Mihalas, 2019), or the effects of injuring mouse networks.

In this study, we build and test Kuramoto oscillator-based models of neurodynamics on a mouse brain connectome (Oh et al., 2014) and examine the key model features that optimize the fit of these models to empirical FC networks from mice (Grandjean et al., 2014). We use the neurodynamics models from this analysis to study how a mouse model of mild TBI (Chen et al., 2014) will affect FC. Our results demonstrate the importance of building subject-specific models of neural dynamics, indicate that key features of the connectome are necessary to generate consistent predictions of changes in neural dynamics after injury, and predict both hypoconnectivity and hyperconnectivity as direct outcomes of TBI.

## METHODS

**Figure 1.**
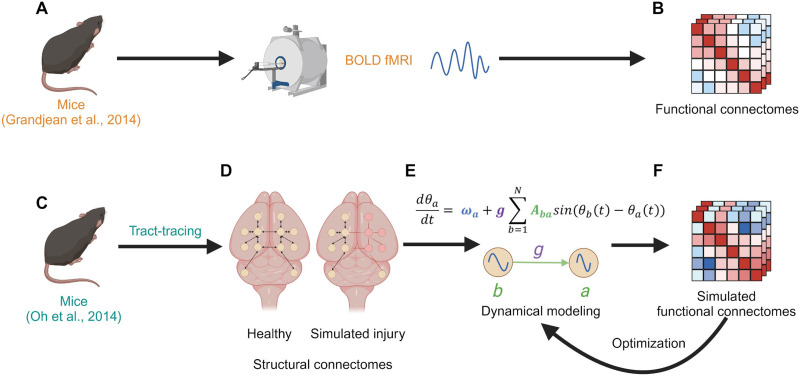
An overview of our modeling methods. Mice were anesthetized and their BOLD signals recorded using fMRI by Grandjean et al. (2014) (A). We used the data from that study which were published in a public dataset (Grandjean, 2020) to generate functional connectomes (B). Tract-tracing experiments (C) were conducted in mice by Oh et al. (2014) and used to generate their directed structural connectivity data (D), which we additionally process into whole-brain directed and undirected SCs. We can also simulate injury by reducing the edge connectivity values for subsets of each connectomes’ regions of interest to explore the effects of injury on our subsequent modeling pipeline. We apply a mathematical dynamics model, the Kuramoto model of coupled oscillators (Kuramoto, 1984) (E), to simulate neural synchronization on these structural networks. From these data, we produce simulated functional connectomes (F). These connectomes can be further used to iteratively optimize our simulations for improved similarity to the empirical functional data. Created in BioRender. Rayfield, A. (2024) https://BioRender.com/z37q721.

In this study, we applied network theory and computational modeling to mathematically model different functional states of the mouse brain from both uninjured and injured connectomes (Figure 1). Our overarching goal was to develop models that were relatively similar to empirical mouse FC and would be disrupted consistently in a dose-dependent manner by a network model of TBI. We used a previously published mesoscale mouse brain connectome (Oh et al., 2014) to represent brain structure and a dynamical model to simulate FC. We compared our simulations with empirical mouse FC networks developed from different anesthetized BOLD fMRI recordings (Grandjean et al., 2014), used an optimization algorithm to more closely fit our model to the empirical data, and explored the influence of injuring the structural network on our simulated FC by decreasing structural edges that were connected to brain regions known to be injured in a mouse model of mild TBI (Chen et al., 2014).

### Mouse Connectome

For our dynamical modeling, we generated a mouse brain SC from Oh et al.’s (2014) best fit, weighted, directed connectivity model of 213 regions of the Allen Mouse Brain Connectivity Atlas (AMBCA) based on their anterograde tracing data. We used an approach from research by Melozzi et al. (2017) and assumed bilateral symmetry, as diffusion MRI tractography has previously observed high similarity between right and left hemisphere white matter tracts in the mouse brain (Calabrese et al., 2015). This method produced a 426-node SC where all ipsilateral and contralateral connection weights directed from the left hemisphere brain regions are equal to their counterparts directed from right hemisphere regions. To explore the effects of using a directed connectivity model more closely, we also developed a weighted and undirected connectivity model from these data, where relative connection weights between each pair of regions were set by adding the connectivity strengths in both directions together. Before simulating dynamics using either network, all connection weights were rescaled to set the maximum edge strength in the network to 1, and in accordance with standard network analysis practices, the main diagonal connections (i.e., self-connections) were set to 0.

### FC From Empirical Mouse fMRI Data

We generated FC networks from publicly available resting-state BOLD fMRI data (Grandjean, 2020) originally collected as part of a single mouse study by Grandjean et al. (2014), which compared the effects of different anesthetics and protocols on resting-state mouse fMRI. We used data from 58 available subjects, which were scanned in groups of 4–14 mice per anesthetic protocol (Table 1). The BOLD time-series data from each mouse were recorded from 604 brain regions of interest. To create FC networks that we could robustly compare with our model predictions, we reduced the 604 regions of interest to 354, which were exact regions in the 426-region SC developed for the computational model, then further chose to include only regions above the median voxel count, using 178 regions. Then, for each individual mouse recording, we further controlled the quality of our analyses by eliminating regions with a BOLD time-series coefficient of variation less than 10^−6^, which enabled us to test the effects of varying the available regions of interest on the accuracy of our predictions during optimization while avoiding regions with unreliable data. This particular quality control step produced a range of sizes for empirical FC networks dependent on the number of excluded regions. Pearson correlations between the BOLD time-series for regions that remained after quality control were used to compute empirical FC networks, resulting in network sizes of 115–146 nodes after quality control.

**Table 1.** List of fMRI samples from different anesthetic conditions and animals recorded by Grandjean et al. (2014)

Anesthetic condition	Abbreviation	Animal sample numbers
Isoflurane 1%	Iso1	0288, 0289, 0290, 0291, 0292, 0293, 0295, 0296, 0297, 0298
Isoflurane 1.5%	Iso1.5	0285, 0286, 0287, 0294
Medetomidine 0.1-mg/kg bolus and 0.2-mg/kg/h infusion	Med0.1	0299, 0300, 0301, 0302, 0303, 0304, 0305, 0306, 0307, 0308, 0309
Medetomidine 0.05-mg/kg bolus + 0.1-mg/kg/h infusion	Med0.05	0310, 0311, 0312, 0313, 0314
Medetomidine 0.05-mg/kg bolus + 0.1-mg/kg/h infusion and isoflurane 0.5%	MedIso	0315, 0316, 0317, 0318, 0319, 0320, 0321
Propofol 30-mg/kg bolus + 120–150-mg/kg/h infusion	Pro30	0322, 0323, 0324, 0325, 0326, 0327, 0328
Urethane 1.5 g/kg intraperitoneal	Ure1.5	0329, 0330, 0331, 0322, 0333, 0324, 0335, 0336, 0337, 0338, 0339, 0340, 0341, 0342

Animal sample numbers are shown as they were listed in a larger, multistudy dataset (Grandjean, 2020).

When comparing FC networks, we identified the common regions of interest to use for each individual comparison and computed the Pearson correlation between the two networks using only the undirected edge strengths between those regions. To assess the similarity of each FC network to their average, we identified 111 regions of interest that were common to all 58 samples, produced an average FC network containing those regions, and then computed the Pearson correlation between all edge strengths from the average FC and the corresponding edges’ strengths from each individual FC network.

### Dynamical Modeling and Simulated FC

We used the Kuramoto model of coupled oscillators to simulate the synchronization of slow oscillations in the BOLD frequency range on the mouse brain SC. Oscillator phases were calculated by numerically solving a system of differential equations:

$$\frac{d\theta_{a}}{dt}=\omega_{a}+g\sum_{b=1}^{N}A_{ba}\sin\left(\theta_{a}-\theta_{b}\right)a=1,2,\ldots ,N$$

where the instantaneous phase of activity at each oscillator *a, θ_a_*, is simulated for *N* = 426 nodes corresponding to each region of the full SC, *A* (Hellyer et al., 2015; Schmidt et al., 2015). Nodes were assigned natural angular frequencies, *ω_a_*, from the BOLD frequency range, specifically the uniform distribution [0.01, 0.1] Hz multiplied by 2*π* (Alderson et al., 2018; Cocchi et al., 2016; Gollo et al., 2017). Initial values for the phase of each oscillator were distributed randomly and uniformly between 0 and 2*π*. The weighted structural connectivity edges *A*_*ba*_ provided the connectivity between each pair of nodes on the SC network, directed specifically from oscillator *b* to oscillator *a* in simulations where the directed network was incorporated. The sum in each equation therefore describes the influence of every other oscillator in the network, *b* = 1, 2, …, *N*, on oscillator *a*. The effects of all connectivity weights are scaled by a global coupling strength constant, *g*. We ran the model across a range of coupling factors and computed the global synchrony, metastability, and predictive power for both the directed and undirected connectomes similarly to prior work (Cabral et al., 2011; Wu et al., 2022).

To investigate the model prior to optimization, we conducted 25 ten-minute simulations for each value of *g*, which was explored (*g* = 0.1–2 for the undirected network and *g* = 0.1–4 for the directed network, with a resolution of 0.1), initialized using 25 random distributions of *ω* and initial *θ* values, and eliminated the first 2 min of each simulation to avoid transient effects. Average global synchrony and metastability were calculated as in prior work using the mean and standard deviation, respectively, of the Kuramoto order parameter *R*(*t*), calculated as:

$$R\left(t\right)=\left|\frac{1}{N}\sum_{b=1}^{N}e^{i\theta_{b}\left(t\right)}\right|$$

where *i* is the imaginary unit and *θ_b_*(*t*) is the instantaneous phase of oscillator *b* among the *N* = 426 total network nodes (Cabral et al., 2011; Hellyer et al., 2015; Wu et al., 2022). To create predicted FC matrices, we calculated *sin*(*θ*) for each oscillator as a simple function of phase to represent neural activity fluctuations (Cabral et al., 2011) and computed FC as the pairwise Pearson correlation of *sin*(*θ*) among all oscillators. The *predictive power* of a simulation was defined as the Pearson correlation between edge strengths from the simulated FC network and the corresponding edge strengths from the empirical FC network (Melozzi et al., 2019).

### Random Connectivity Architectures

To determine if our chosen AMBCA-based architecture influenced the predictive power of these initial simulations, we produced a random, directed connectivity architecture from the directed network. We applied an algorithm from the Brain Connectivity Toolbox (Rubinov & Sporns, 2010), which randomly swapped each edge in the network an average of 20 times while preserving the distributions of edge weights directed in and out of each node (Rubinov & Sporns, 2011). We also generated a corresponding undirected model by adding the edge weights in both directions between each pair of nodes together. We repeated our procedures for simulation using the same set of *ω* distributions and random initial *θ* values for the appropriate range of *g* values (0.1–2 for the undirected network, 0.1–4 for the directed network) and determined the average predictive power for the randomized models, directed and undirected, at each coupling strength. We compared the peak predictive powers for each empirical FC network with those previously computed in the original, nonrandomized simulations.

### Optimizing the Dynamical Model’s Predictive Power

To improve our simulations’ predictive power and account for differences among individual mice subject to various anesthetic protocols (Grandjean et al., 2014), we developed an optimization algorithm to better match simulated FC to any target empirical FC network. To this end, we conducted 100 iterative dynamics simulations per empirical FC network (Supporting Information Figure S1), during which the size of the SC (*N*) was reduced in each simulation to include only nodes that were present in the target empirical FC network of interest, unlike in our original simulations, so that all simulated nodes could be compared with the target data and controlled. We used global coupling strengths that produced approximate maximum metastability for the reduced SC—proposed as the point where the dynamics of a network are most flexible—*g* = 1.0 for undirected and *g* = 3.5 for directed (Supporting Information Figure S2). After each 200-s iteration, we updated each node’s natural frequency by an increment scaled to the initial frequency range by a constant, 0.005, and directly proportional to the node’s control energy (*E*_*a*_) and the difference between the node’s strength (the sum of positive functional network edges involving that node) on the simulated FC and on the empirical FC. Control energy for a node *a* was calculated as:

$$E_{a}=\int_{0}^{T}{\left|u_{a}^{*}\left(t\right)\right|}^{2}dt$$

where u* is the optimal control input calculated for a linear approximation of brain dynamics:

$$\frac{dx\left(t\right)}{dt}=x^{\prime }\left(t\right)=Ax\left(t\right)+Bu\left(t\right)$$

where ***x***(*t*) describes the total edge strength for each FC node, ***A*** is the stabilized structural connectivity matrix, ***B*** is an identity matrix of equal size, and the initial state and target states for ***x***(*t*) were chosen to be the simulated FC network and the empirical FC network, respectively (Braun et al., 2021; Gu et al., 2015). All initial simulations used the same random natural frequencies for specific nodes as we determined that the variation in optimal predictive power between simulations targeting dissimilar empirical FCs was not substantially affected by initial conditions (Supporting Information Figure S3). Additional simulations showed the optimized predictive power was affected by low coupling strength on the directed models (Supporting Information Figure S4). For this reason, the approximate coupling strengths for maximum metastability were maintained for the reduced-size undirected and directed networks. As frequencies higher than 0.1 Hz have previously been detected from the BOLD signal using fast fMRI (Lewis et al., 2016), we did not exclude optimized distributions with frequencies greater than 0.1 Hz.

### Modeling Brain Injury Using Connectivity Reduction

To explore the dynamical model’s sensitivity to injury, we chose to reduce the strength of structural connectivity edges connected to regions that are injured in a mouse model of mild TBI: mild controlled cortical impact (mild CCI; Chen et al., 2014). As mild CCI produced ipsilateral neurodegeneration that had previously been measured by Fluoro-Jade B staining (Chen et al., 2014), we developed a list of 14 cortical and hippocampal regions on our connectome by locating regions on the Allen Mouse Brain Atlas, which approximately overlapped the staining pattern, as viewed on the Scalable Brain Atlas using version 3 of the Common Coordinate Framework (Bakker et al., 2015; Lein et al., 2007; Wang et al., 2020). We generated 20 additional connectomes per optimized model where all edge strengths directed to and from these regions of interest for the first hemisphere in the connectome were decreased in increments of 5% of their maximum value, analogous to the process of incrementally removing neurons from computational neuronal networks to model damage (Gabrieli et al., 2020; Schumm et al., 2020). Thus, these connections lost between 5% and 100% of their original connectivity to represent variable severities of neuronal injury. Global efficiency on the original and injured structural networks was calculated as the average inverse of the shortest path length between any two nodes on the network (Rubinov & Sporns, 2010), where path length of each edge weight on the network was defined as the inverse of its connection strength.

We ran FC simulations for each network subject to connectivity reduction based on our optimized models of both directed and undirected connectivity. For each of these simulations, we could only decrease connectivity at eight or nine regions, which were present in the corresponding SC reduced to fit a specific empirical FC.

### Determining Changes in FC Strength After Injury

To further quantify the changes in simulated FC after injuring each optimized model, we calculated the changes in FC strength at nodes in the whole-brain network. Strengths were calculated using nodes common to all 58 optimized models and excluding any directly damaged regions. We again computed nodal FC strength as the sum of all positive FC edge weights connected to a node (Rubinov & Sporns, 2010). To explore the heterogeneity in postinjury outcomes among our optimized models, we reported frequency distributions for 103 regions of interest common to all optimized connectomes, describing the proportion of the 58 models for which a region’s strength decreased (i.e., for which the nodal FC strength for the uninjured network was greater than the nodal strength for the injured network) at a given injury level.

### Statistical Analysis

Comparisons between specific pairs of groups were conducted using the two-sample *t* test. For comparisons among multiple groups, the significance of any differences among the group means were determined using one-way analysis of variance (ANOVA) and followed by Tukey’s multiple comparisons test to determine if specific pairs of group means were significantly different; if variances among groups were unequal by the Brown-Forsythe test, Welch’s ANOVA and Dunnett’s T3 multiple comparisons test were used instead. We used the Pearson correlation coefficient to examine the association between pairs of variables, and applied the Kolmogorov-Smirnov (K-S) test to compare the frequency distributions of FC strength decrease at different injury levels. We used the standard *α* = 0.05 threshold for *p* value significance throughout this study.

## RESULTS

### Empirical Mouse fMRI Data Were Variable, Even Within Anesthetic Groups

**Figure 2.**
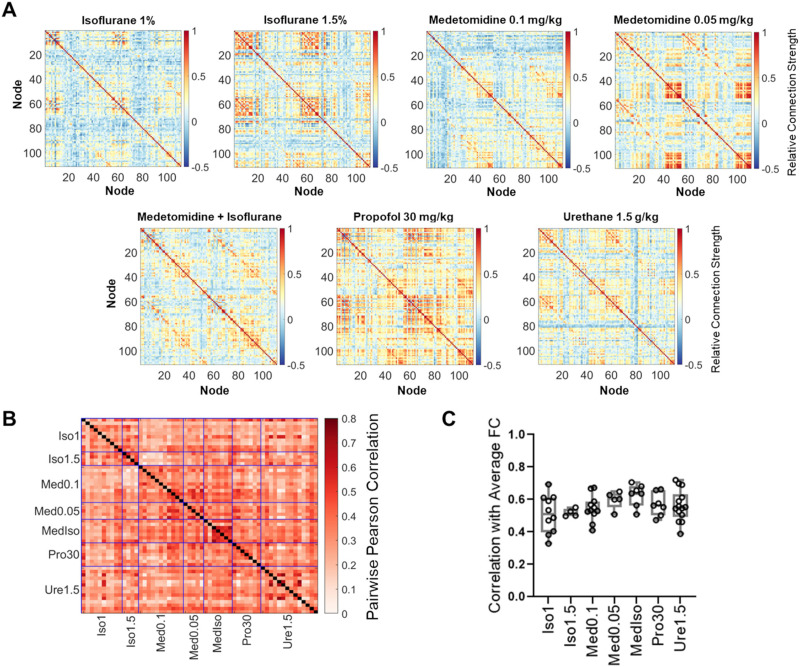
FC heterogeneity observed after processing BOLD fMRI data obtained from resting-state, anesthetized mice by Grandjean et al. (2014) into FC networks. (A) Representative FC networks from mouse data collected under various anesthetic conditions, individually processed from the original data (Grandjean et al., 2014), visualized using 111 common regions of interest. (B) Pairwise Pearson correlations between the common edges of 58 individual FC networks categorized into different anesthetic conditions. (C) Group-level comparison of the Pearson correlation of each FC network to the average FC network. There was no significant effect of the anesthetic group on similarity to the average (ANOVA *p* = 0.16, ns), and the mean correlation with the average FC within each group varied between 0.51 (Isoflurane 1%) and 0.63 (medetomidine + isoflurane). Refer to Table 1 for a comprehensive overview of each anesthetic protocol as described by Grandjean et al. (2014), their abbreviations, and the number of mice per group.

Before developing simulations of mouse FC, we first examined the consistency of the empirical FC within a group of animals and compared the FC within and between anesthetic states (Figures 2A and 2B). We sought to test how animal-to-animal variance compared with the differences that could exist across different anesthetic states and inform our model development. As a reference, we computed an average FC across all animals (*n* = 58). Correlations of each empirical FC network with this reference FC network showed that considerable variation existed within anesthetic groups; only 16% of the variance could be attributed to the different types and depths of anesthesia (ANOVA group sum of squares (SS) = 0.072, total SS = 0.45). In turn, we did not observe a significant influence of anesthesia on the correlation of empirical FCs to the reference (ANOVA *p* = 0.16, ns; Figure 2C). Based on these results, we concluded that we needed to optimize the computational model parameters for each individual animal and could not use an averaged FC matrix for all mice or each anesthetic state.

### Initial Simulations on the Directed Connectome Predicted the Empirical Data Better Than Those on the Undirected Network

After examining the consistency of the measured FCs across anesthetic groups, we developed Kuramoto oscillator-based models for predicting mouse brain dynamics in each animal. The first feature studied in our model was the connectivity architecture used to estimate neural dynamics. Most prior work assigned equal weights for network edges into and out of each node (i.e., an undirected network). In comparison, different weights for input or output edges (i.e., a directed network), which can be assigned using mouse brain tract-tracing data, are used much less frequently (Choi & Mihalas, 2019).

**Figure 3.**
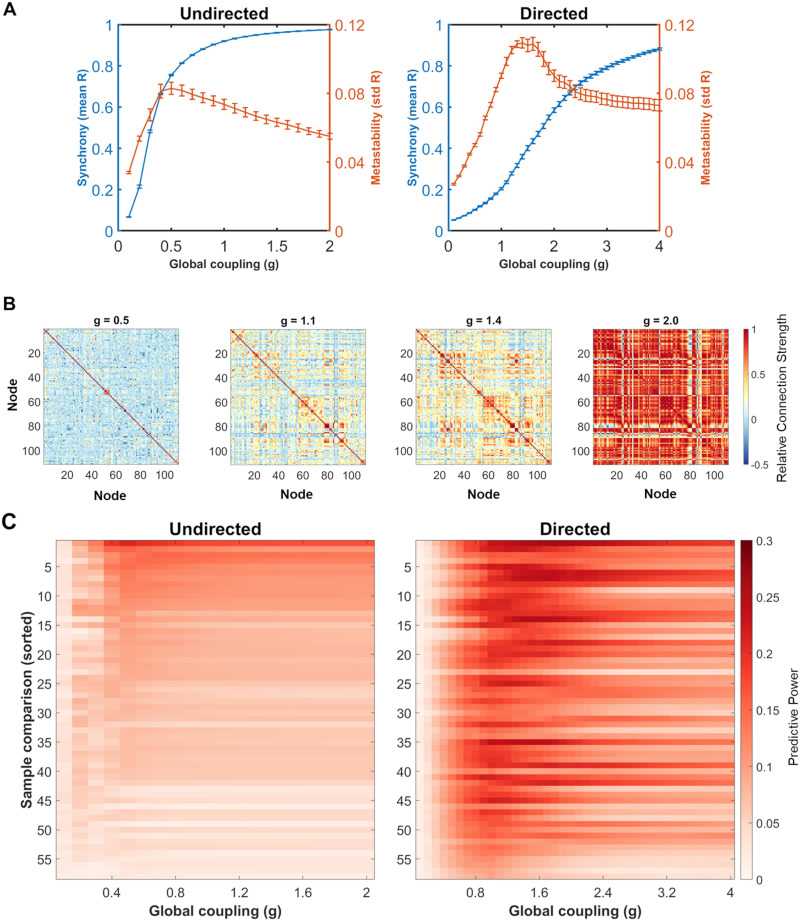
The undirected and directed mouse brain connectivity display different Kuramoto model dynamics and baseline similarity between the simulations and empirical data. (A) Differences between undirected and directed network connectivity when simulating neural synchronization on the Kuramoto model and varying the global coupling strength (*g*) for 25 randomized initial conditions and frequency distributions. Maximum metastability occurs for the undirected network at *g* = 0.5 and for the directed network at *g* = 1.4. (B) Example simulated FC matrices generated by the pairwise correlation of *sin*(*θ*) from directed Kuramoto oscillators, using the same initial conditions (random initial phase distributions and natural frequency distributions) for each representative coupling strength (*g* = 0.5, 1.1, 1.4, and 2.0) and displaying the 111 common regions of interest used in Figure 2. (C) Predictive power of simulated FC from the Kuramoto model over a range of global coupling strength values for 58 empirical FC derived from resting-state, anesthetized mouse BOLD fMRI data (Grandjean et al., 2014), determined for both the undirected (left) and directed (right) networks. Each value of predictive power for a specific *g* value is an average correlation between a simulated FC matrix and the empirical FC sample for the same 25 sets of initial simulation conditions. Comparisons with samples are sorted from 1 to 58 by decreasing average maximum predictive power of the undirected simulations to the sample. The best predictive power of a simulated FC network from our initial Kuramoto model for an empirical FC network was significantly improved by the use of directed connectivity (average predictive power = 0.085 for undirected, average predictive power = 0.18 for directed, *p* < 0.0001).

The choice of directed or undirected architecture affected the typical measures of Kuramoto oscillator dynamics: synchrony and metastability. In general, both directed and undirected architectures showed an ability to synchronize and to produce a maximum point of metastability (Figure 3A). The coupling strength needed to maximize metastability was higher for the directed network (*g* = 1.4) than the undirected network (*g* = 0.5), and when comparing the undirected and directed networks’ dynamics at *g* = 1.4, the mean synchrony and metastability values were both significantly different (mean undirected synchrony = 0.96, mean directed synchrony = 0.36, *p* < 0.0001; mean undirected metastability = 0.065, mean directed metastability = 0.11, *p* < 0.0001). Varying the coupling strength also affected simulated FC (Figure 3B).

We next optimized the coupling strength of each model type to best predict the 58 empirical FC networks. Adjusting the coupling strength is a common approach to achieve better correlation between modeling and experimental FC data (Cabral et al., 2011; Wu et al., 2022). To this end, adjusting the coupling strengths of the model substantially improved the average predictive power of the model for the 58 empirical FC network in the dataset (Figure 3C). Simulations using the directed connectivity architecture predicted the data significantly better than undirected connectivity, but neither were strongly correlated with the measured data (undirected model average predictive power = 0.085, directed model average predictive power = 0.18, *p* < 0.0001).

### Brain Architecture Is an Important Component of the Kuramoto Model

**Figure 4.**
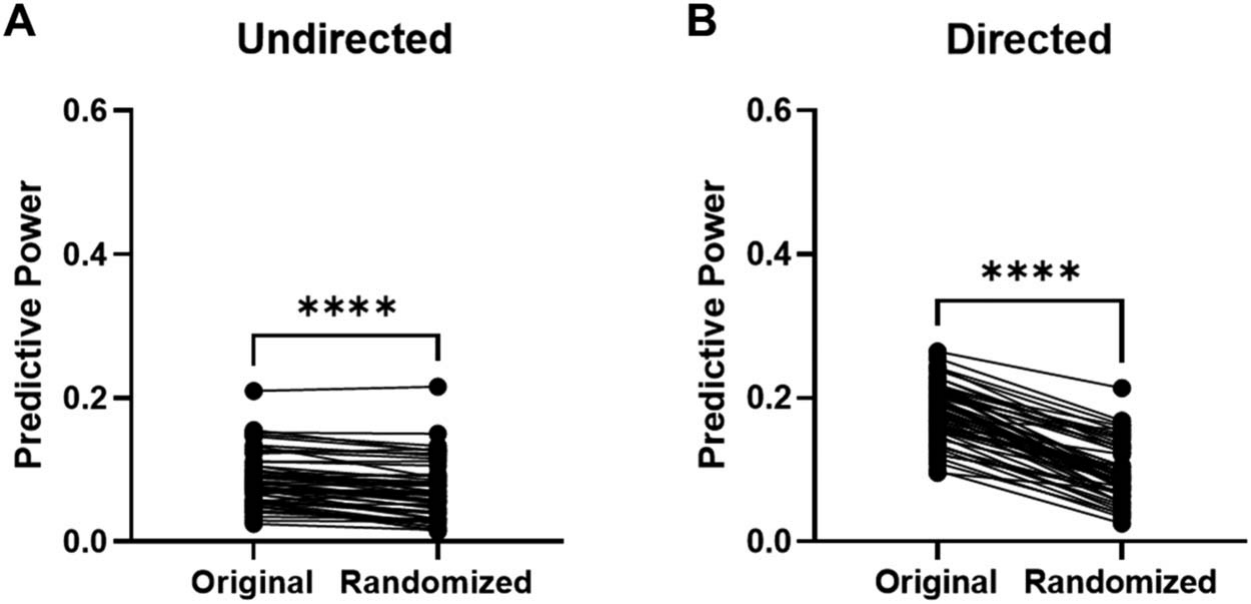
The predictive power for averaged comparisons with each of the 58 empirical FC was significantly higher when using the original connectivity models than randomized connectivity models. (A) A paired *t* test demonstrates that the predictive power of comparisons were significantly decreased by randomization (randomized − original predictive power = −0.016), and the difference in mean predictive power was significant by the unpaired *t* test (*p* = 0.023). (B) A paired *t* test demonstrates that the predictive power of comparisons were significantly decreased by randomization (randomized − original predictive power = −0.086), and the difference in mean predictive power was significant by the unpaired *t* test (*p* < 0.0001). (****p < 0.0001 for paired *t*-test results).

After identifying differences in predictive power between simulations using undirected and directed connectivity, we sought to characterize the influence of the AMBCA structural architecture on our simulations’ predictions. We produced a random directed architecture that preserved the distribution of in-strength and out-strength from the directed AMBCA network, summed the directed edges together to produce a corresponding random undirected architecture, and compared the peak predictive power achieved through simulations on these networks with the peak predictive powers we reported using the original architectures. Broadly, we found that randomization reduced the peak predictive power of each prediction, with a greater effect on the directed simulations. The random undirected architecture produced simulations with reduced peak predictive power relative to the undirected AMBCA-based network (Figure 4A), with the relatively small difference (random − undirected = −0.016) demonstrating significance during unpaired *t* testing (*p* = 0.023) and paired *t* testing (*p* < 0.0001), confirming that the optimal comparisons between the simulations to specific empirical FC networks were significantly reduced by randomization. This finding was also clear for simulations on the directed AMBCA-based network when compared with the random directed network (Figure 4B), with a larger difference in predictive power (random − directed = −0.086), which was highly significant for unpaired (*p* < 0.0001) and paired (*p* < 0.0001) *t* testing. Due to the significance of this influence on our initial simulations, for the remainder of the study, we used the nonrandomized network architectures.

### Frequency Optimization More Accurately Models FC for Both Undirected and Directed Simulations

**Figure 5.**
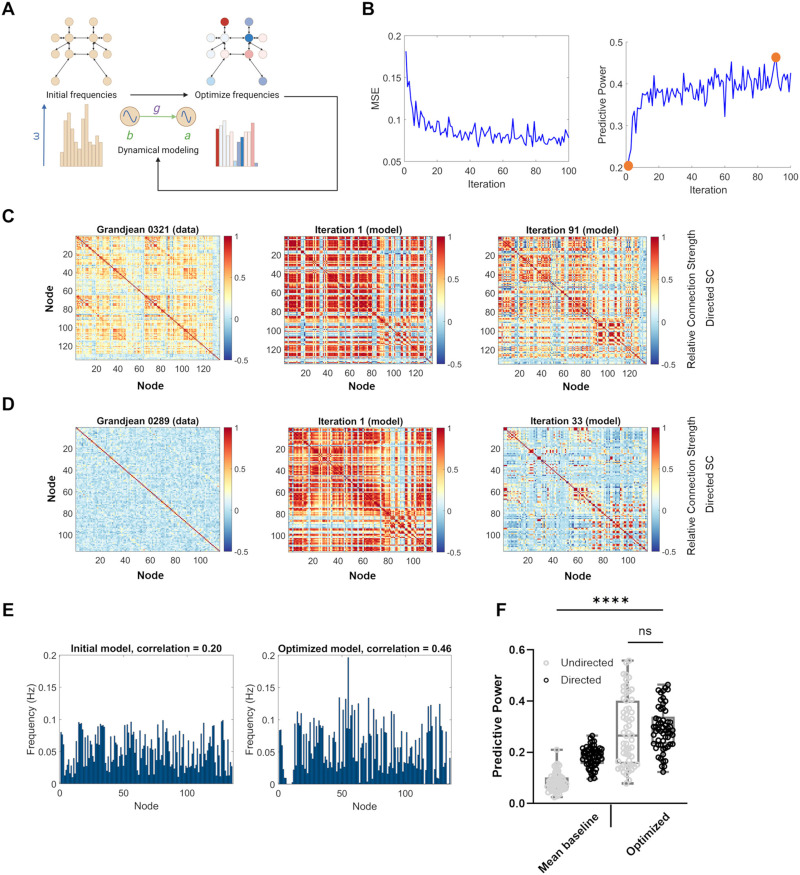
The influence of natural frequency seeding on our model’s results provides an opportunity for frequency optimization to better predict empirical data. (A) Starting from a single, random natural frequency distribution like those we have previously used, we implemented an iterative algorithm to optimize the frequency distribution to match a specific target brain state. Frequencies were updated after comparison of simulated FC with the target data, and then the model was used again for up to 100 iterations. Created in BioRender. Rayfield, A. (2024) https://BioRender.com/x02x666. (B) Mean squared error (MSE) and predictive power for a single instance of the optimization algorithm applied to the directed network for 100 iterations, using a MedIso sample (0321) as target data. Each metric is used to compare the simulated data with the empirical data. The first iteration and iteration with peak predictive power (iteration 91) are circled in orange. (C) Empirical FC network derived from the online dataset by Grandjean (2020) for which the model achieved the best optimal predictive power (sample 0321, *R* = 0.46 at iteration 91; left), compared with the first simulation (center) and the optimal simulation (right). (D) Empirical FC network for which the model achieved the worst optimal predictive power (sample 0289, *R* = 0.12 at iteration 33; left), compared with the first simulation (center) and the optimal simulation (right). (E) Initial natural frequency was randomly distributed in the [0.01, 0.1] Hz range, and the optimal frequency distribution arrived during the same algorithm instance portrayed in B. (F) When comparing the mean predictive power achieved between the baseline simulations and the 58 empirical FC network (Figure 4) with the optimal predictive power achieved for each FC network by the optimization algorithm, we find that the average correlation by sample is affected by the simulation method (Welch’s ANOVA, *p* < 0.0001). (Average predictive powers: mean baseline undirected = 0.085, mean baseline directed = 0.18, optimized undirected = 0.28, optimized directed = 0.29.) The comparison between optimized, undirected correlations and optimized, directed correlations was the only comparison among this group to not be statistically significant during post hoc testing (*p* = 0.9990; ****p < 0.0001).

Another modeling parameter that can influence the predicted brain dynamics is the distribution of natural frequencies assigned to each node. To our knowledge, this model feature is less commonly examined to resolve discrepancies between modeling and experimental FC data. However, past theoretical work studies how adjusting the natural frequencies of individual nodes of brain networks, including during dynamical simulations, may trigger the transition of network activity to different states, which may have applications for steering the brain toward more flexibility or away from pathological states (Braun et al., 2021; Gu et al., 2015; Muldoon et al., 2016). Based on this prior research, we implemented an optimization algorithm to adjust the natural frequencies of each node and decrease the error between simulated and empirical FC. Natural frequencies were iteratively updated (Figure 5A) to minimize mean squared error between simulated and empirical data, which consequently improves the simulations’ predictive power (Figure 5B). The optimal predictive power achieved during these iterations varied between samples. For example, the optimal frequency distribution targeted to sample 0321 on the directed connectivity model (Figure 5E) achieved the highest peak predictive power of 0.46 (Figure 5C), while the optimal frequency distribution targeted to sample 0289 achieved the lowest peak predictive power of 0.12 (Figure 5D). Optimization on both the undirected and directed network models achieved higher average predictive power than the baseline simulations, which only used global coupling strength as an optimization parameter (undirected predictive power = 0.28 [frequency optimized] vs. 0.085 [coupling strength optimized], *p* < 0.0001; directed predictive power = 0.29 [frequency optimized] vs. 0.18 [coupling strength optimized], *p* < 0.0001; Figure 5F). Unlike the coupling strength optimization process, which showed a clear advantage of the directed model framework, frequency optimizations led to no significant difference in the optimal fit for directed or undirected models (*p* = 0.9990, ns). However, the dynamical variables previously measured, synchrony and metastability, differed between undirected and directed models after optimization (Supporting Information Figure S5).

**Figure 6.**
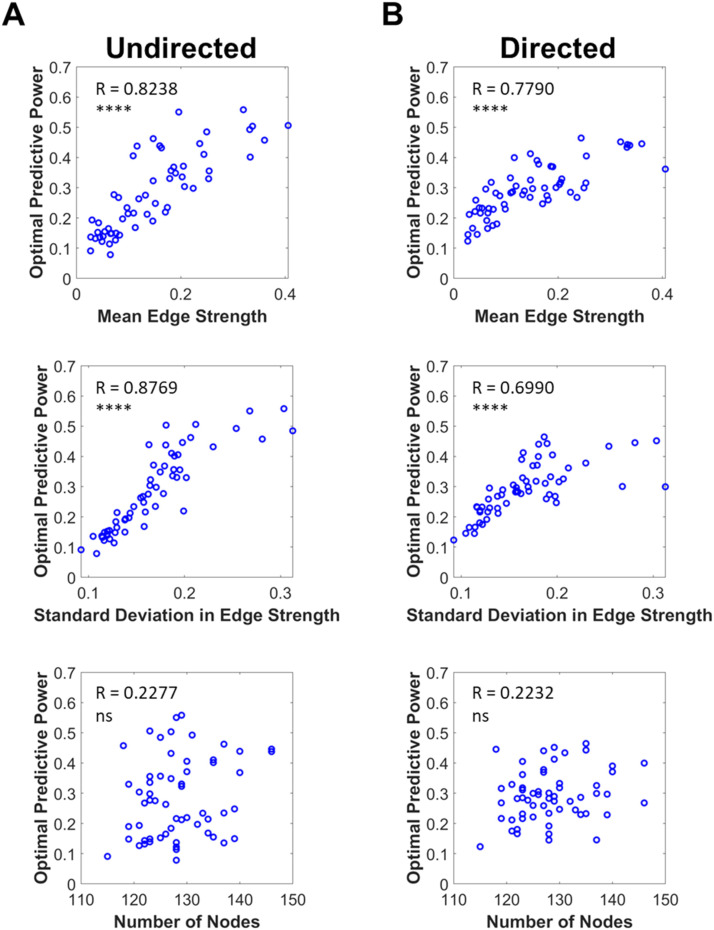
Characteristics of target empirical data were related to the optimal similarity our algorithm could achieve. (A) For the undirected models, optimal predictive power was strongly correlated with mean edge strength (top, *R* = 0.8238) and the standard deviation in edge strength (center, *R* = 0.8769) of the target FC network, but not significantly correlated with the target’s number of nodes (bottom, *R* = 0.2277, *p* = 0.086). (B) The same trends were found for predictions from the directed models. Optimal predictive power was strongly correlated with mean edge strength (top, *R* = 0.7790) and the standard deviation in edge strength (center, *R* = 0.6990) of the target FC network, but not significantly correlated with the target’s number of regions of interest (bottom, *R* = 0.2232, *p* = 0.092; ****p < 0.0001). Substantial differences in optimal predictive power between target FC networks were stable for multiple optimizations (Supporting Information Figure S4; *R*: Pearson correlation).

Our next step was to understand if there were features of the measured FC data, rather than the model itself, which would make a subset of imaging data ideally suited to the Kuramoto model framework. Characteristics of the target data were significantly related to the optimized correlation for both the directed and undirected models. For the undirected model, the mean FC edge strength (*R* = 0.82, *p* < 0.0001) and standard deviation in FC edge strength (*R* = 0.88, *p* < 0.0001) were significantly related to optimal predictive power while the number of regions of interest (*R* = 0.23, *p* = 0.086, ns) was not (Figure 6A). The same trends also influenced the optimal performance of the directed model. Mean FC edge strength (*R* = 0.82, *p* < 0.0001) and standard deviation of FC edge strength (*R* = 0.88, *p* < 0.0001) were significantly related to predictive power of the model while the number of regions of interest (*R* = 0.22, *p* = 0.092, ns) was not (Figure 6B).

In all, these analyses identified several features of Kuramoto models and data that influence the model’s capacity to predict the empirical FC dataset:

- The measured FC has high animal-to-animal variance, more than the effect of anesthesia level and type, which requires one to simulate each animal, rather than groups of animals.
- Although directed structural connectivity provides a better fit to the measured data than undirected connectivity, these differences disappear when the natural frequencies of the nodes are used to optimize the fit of the model to the data.
- Features of the original FC dataset—namely, the average edge strength and variance of the edge strength in the FC matrix—had a strong influence on the fit between the model and the measured data.

### Network Injury Most Consistently Affects Simulated FC From the Directed Model

After comparing the performances of both directed and undirected models, we tested their utility in predicting a dose-dependent response to simulated TBI. Broadly, experimental TBI models in mice often use a focal mechanical insult—such as a fluid percussion pulse or a CCI to the brain surface—to cause histological changes in the brain and cognitive impairments in the animal. The changes caused by CCI are most often modeled with finite element models of the brain (Chen et al., 2014; Pleasant et al., 2011). In general, increasing the mechanical depth of impact and, to a lesser extent, the impact velocity will increase the extent of deformation throughout the cortex and underlying hippocampus. Similarly, past studies show both histological damage and cognitive impairments increase in severity as the depth and velocity of impact are increased (Chen et al., 2014; Pleasant et al., 2011; Saatman et al., 2006).

**Figure 7.**
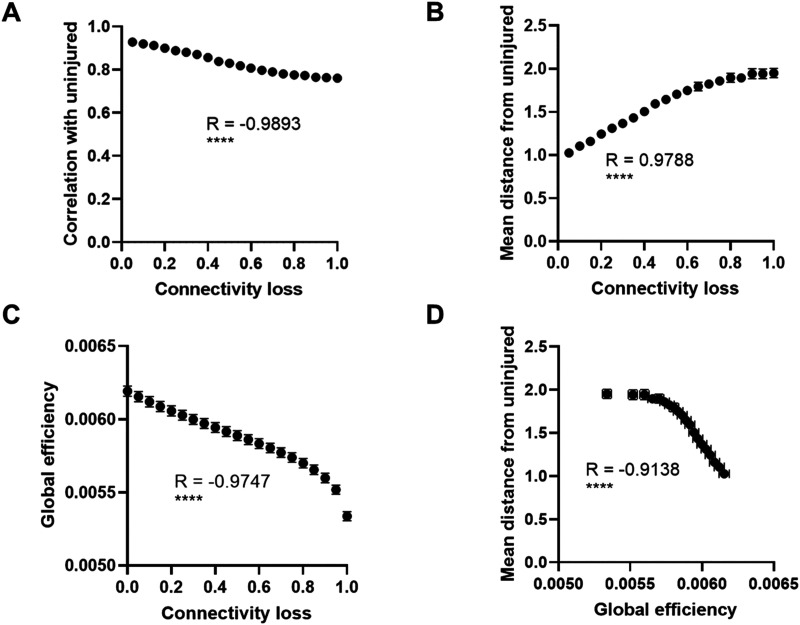
Optimized FC is disrupted by network injury in a select series of brain regions (Chen et al., 2014; Table 2), and this disruption can also be predicted by the change in a whole-network metric: global efficiency. (A) As the fraction of edge connectivity lost for regions of interest injured during mild CCI increased, the average correlation between the simulated FC network and the uninjured, optimized FC network decreased (*R* = −0.9893, *p* < 0.0001). (B) These changes to simulated FC can also be demonstrated by an average increase in the mean regional distance between simulated FC and uninjured FC as the connectivity loss increases (*R* = 0.9788, *p* < 0.0001). (C) Connectivity loss was strongly anticorrelated on average with the global efficiency of each structural network used for optimized simulations (*R* = −0.9747, *p* < 0.0001). (D) The structural networks’ global efficiency and simulated FC distance from the baseline were also strongly anticorrelated on average (*R* = −0.9138, *p* < 0.0001), suggesting further that a brain injury and its effect on FC may be characterized using global efficiency loss as a measure of structural damage. Simulations used in this figure were conducted on the optimized, directed Kuramoto models at their approximate global coupling strength for maximum metastability (*g* = 3.5), data from the 58 optimized models and underlying structural networks were averaged, and the trends observed for individual optimized models can vary (Figure 8; R: Pearson correlation).

To this end, we simulated neural synchronization on our directed, optimized models and reduced connectivity at regions where neurodegeneration in a mouse model of mild CCI was identified at 24 hr (Chen et al., 2014; Table 2). Not knowing the precise relationship between mechanical deformation and connectivity changes, we progressively reduced connections across selected regions on each structural network simultaneously. These changes also carried over to models of FC: increasing the extent of damage via connectivity loss led to a linear reduction in the correlation between injured FC networks and their uninjured, optimized model FC networks from the same modeling conditions (*R* = −0.9893, *p* < 0.0001; Figure 7A), as well as a linear increase in the mean Euclidean distance between injured FCs and the uninjured FCs (*R* = 0.9788, *p* < 0.0001; Figure 7B). This progressive injury model created a significant reduction in global efficiency of the network (*R* = −0.9747, *p* < 0.0001; Figure 7C), reminiscent of changes in global efficiency that may occur in TBI patients (Chung et al., 2019; van der Horn et al., 2017a). This change in global efficiency also strongly predicted the distance between the injured and uninjured FC simulations (*R* = −0.9138, *p* < 0.0001), suggesting that the approach of using global efficiency as a structural predictor could generalize these trends to other injury models (Figure 7D).

**Table 2.** Abbreviations and names for all brain regions that were initially selected for unilateral connectivity reduction based on the Fluoro-Jade B stain observed 24 hr after mild CCI by Chen et al. (2014)

Brain region	Full name
AUDd	Dorsal auditory area
CA3	Hippocampus, field CA3
DG	Hippocampus, dentate gyrus
PTLp*	Posterior parietal association areas
RSPagl*	Retrosplenial area, lateral agranular part
RSPd*	Retrosplenial area, dorsal part
SSp-bfd	Primary somatosensory area, barrel field
SSp-tr	Primary somatosensory area, trunk
SSs	Supplemental somatosensory area
VISal*	Anterolateral visual area
VISam*	Anteromedial visual area
VISl**	Lateral visual area
VISp	Primary visual area
VISpm	Posteromedial visual area

When analyzing smaller networks that were used with the optimization algorithm, only subsets of these regions that were contained in each smaller network could be selected. * = Region excluded from optimized connectomes due to voxel count thresholding; ** = Region excluded from a subset of optimized connectomes due to signal quality thresholding.

**Figure 8.**
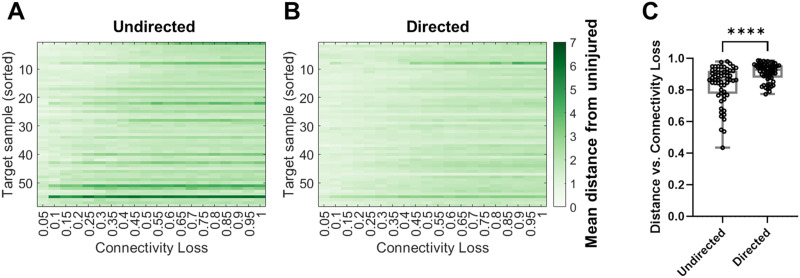
Connectivity injury in a select series of brain regions (Chen et al., 2014; Table 2) disrupts simulated FC for optimized models, particularly for simulations on the directed connectome. Changes in simulated FC can be measured using the distance between simulated FC and the uninjured FC, which increases in response to reduced connectivity for (A) the optimal undirected models (average distance vs. connectivity loss = 0.84) and for (B) the optimal directed models (average distance vs. connectivity loss = 0.91). The average distance versus connectivity loss relationship was significantly more correlated for the directed models (C), suggesting that simulations on the directed connectome respond more consistently to network injury than those on the undirected connectome (****p < 0.0001). Comparisons with samples in A and B are sorted from 1 to 58 by decreasing correlation between each of the mean distance from uninjured network and connectivity.

To compare the effects of injury on our different connectivity models, we next injured the 58 undirected, optimized models as well. The mean Euclidean distance between injured and uninjured FC simulations increased linearly with an increasing loss of connectivity when using both the undirected structural network (Figure 8A) and the directed structural network (Figure 8B). The sensitivity of the optimized models to injury (i.e., best fit slopes of distance vs. connectivity loss) did not significantly differ between undirected and directed models. However, when we examined the correlation between this mean Euclidean distance measure and the degree of remaining connectivity after injury for individual models and compared the results of using each connectivity type, we found that the correlation between distance and connectivity was stronger for directed network simulations than undirected network simulations (*R* = 0.91 for directed, *R* = 0.84 for undirected, *p* < 0.0001; Figure 8C), suggesting that the observed dose-dependent response to injury is more robust when simulating FC using the directed network, though this finding is sensitive to the target FC of each optimized model and the global coupling strength (Supporting Information Figure S6).

### Simulated Network Injury Produces Both Increased and Decreased Regional Nodal Strength on Simulated FC Networks

**Figure 9.**
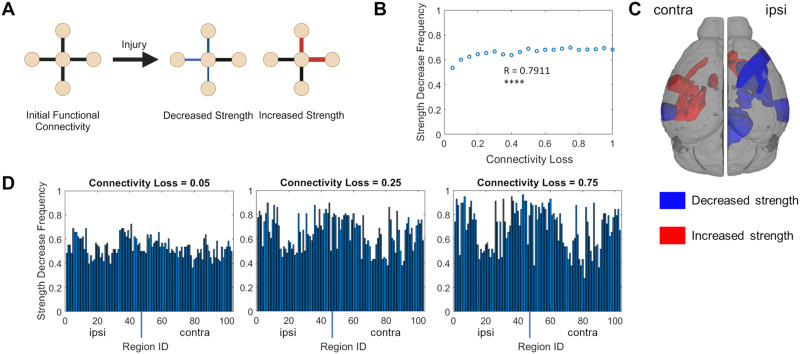
FC changes after simulated injury involve both increases and decreases in FC edge strength. (A) Strength at a network node is calculated as the sum of all FC edges connected to that node. After an injury, we can determine whether the FC strength at a specific node has decreased or increased. Created in BioRender. Rayfield, A. (2024) https://BioRender.com/m36a192. (B) We considered only the FC edges between 103 nodes corresponding to regions of interest that were common to all 58 optimized models and not directly injured by the connectivity reduction model. Among all these nodes in our optimized models, nodes decreased in strength more frequently after injury as the connectivity loss fraction increased (*R* = 0.7911, *p* < 0.0001). (C) Visual representation of the considered brain regions that most frequently displayed a decrease in FC strength (blue) and that most frequently displayed an increase in FC edge strength (red) after a single injury level (connectivity loss = 0.75). Broadly, the brain regions that most frequently displayed a strength decrease were ipsilateral to the injured nodes, while those that most frequently displayed a strength increase were contralateral. Refer to Supporting Information Table S1 for a list of the visualized brain regions. Created with DSI Studio. (D) The frequency distribution of strength decreases after minimal injury (left, connectivity loss = 0.05) was significantly different from the distribution at a more severe injury (center, connectivity loss = 0.25, K-S *p* < 0.0001), which also significantly differs from the distribution at a relatively severe injury level (right, connectivity loss = 0.75, K-S *p* = 0.015; *R*: Pearson correlation).

To broadly compare the results of our optimized, injured simulations to existing literature regarding FC and rodent TBI, we examined whether different nodes’ FC strengths were decreased or increased by TBI. Previous findings in humans and rodents have suggested that both decreased functional connection strength (“hypoconnectivity”) and increased functional connection (“hyperconnectivity”) can occur after TBI (Figure 9A) and that these effects may be distributed in different brain regions or functional subnetworks (Kulkarni et al., 2019; Zhou et al., 2012).

When considering all nodes that were common to our 58 optimized models and not directly part of the regions directly targeted by our connectivity loss model of injury, we found that the simulated FC strength was more likely to decrease at any specific node as the severity of the simulated injury increased (i.e., as the fraction of remaining connectivity strength decreased; *R* = 0.7911, *p* < 0.0001; Figure 9B). The specific brain regions that most frequently displayed a decrease in FC strength across our models tended to be in the ipsilateral hemisphere and included both cortical and subcortical regions, while the regions that most frequently displayed an *increase* in FC strength included ipsilateral regions involved in scent processing (the olfactory tubercle and the taenia tecta) and contralateral regions, particularly regions in the contralateral amygdala (Supporting Information Table S1; Figure 9C). The nodal distribution of FC strength decrease frequencies among our models were noticeably different between specific injury levels; for example, the nodal distribution of strength decrease frequency at minimal injury (i.e., connectivity loss = 0.05) differed from the nodal distribution strength at slightly more severe injury (connectivity loss = 0.25; K-S *p* < 0.0001), but the distribution between connectivity loss = 0.25 and connectivity loss = 0.50 was not different (K-S *p* = 0.11, ns; Supporting Information Figure S7), and this general lack of difference persisted between more severe injury levels (Supporting Information Figure S7). Overall, these simulated FC results demonstrate the potential presence of both hypoconnectivity and hyperconnectivity for the functional connections at different brain regions as a consequence of connectivity loss, even at severe injury levels, though increased severity does lead to relatively more common regional hypoconnectivity throughout our simulated outcomes.

## DISCUSSION

Our broad goal was to develop optimized computational models of mouse brain dynamics that would be disrupted by a realistic injury, allowing us to explore possible FC changes after experimental TBI. We found that structural directionality and a new fitting process that adjusted the frequencies of each node contributed to our models’ predictive power. The optimized models predicted a consistent magnitude of FC disruption in response to a degree of simulated mild CCI, a model of mild TBI. Intriguingly, the models predicted regions of increased connectivity (hyperconnectivity) and decreased connectivity (hypoconnectivity) after injury, resembling the changes observed in FC after both experimental and human TBI.

One of our first steps was analyzing whether the experimental data showed enough similarity within anesthetic states to develop generalized models for each. Past work has commented on differences among mouse anesthetic states; for example, Grandjean et al. (2014) noted that their propofol and urethane procedures produced relatively shallow and deep anesthesia, respectively, and that medetomidine anesthesia could produce epileptic activity when not combined with isoflurane. Likewise, mouse fMRI collected under a combination of low-dosage medetomidine and isoflurane may be optimal for consistently measuring resting-state FC patterns such as cortical and striatal connectivity (Fukuda et al., 2013; Grandjean et al., 2014, 2020). However, we did not find significant differences among anesthetic groups’ similarity to the average FC network. Without a rationale for averaging animals within each anesthetic group, we generated best fit models to each of the 58 measured FC networks and used these models to examine a range of potential healthy and injured brain states.

Our model is a simplified representation of brain dynamics, and this will inevitably limit the predictive accuracy of the model until its predictions are rigorously tested. The Kuramoto oscillator model’s simplified formulation has both advantages and disadvantages—the low computational complexity can provide insights into how manipulations at specific brain regions affect global brain dynamics (Gollo et al., 2017; Schmidt et al., 2015; Váša et al., 2015) without great computational cost. To this end, the Kuramoto model has been used to predict how FC may arise from brain structure in health or human TBI (Cabral et al., 2011; Hellyer et al., 2015; Wu et al., 2022) and can simulate FC with fidelity comparable with other neurodynamics models (Messé et al., 2015). However, one design limitation is determining the ideal pattern of natural frequencies to distribute throughout the network. Our work uses random oscillations in the BOLD range similarly to how slow oscillations have been used in prior research (Alderson et al., 2018; Cocchi et al., 2016; Gollo et al., 2017; Schmidt et al., 2015) and then optimizes their frequencies and computes FC directly from correlations in the Kuramoto output. An alternative choice was to use nodal frequencies from higher frequency EEG bands, such as gamma oscillations, which may produce substantially different results from the low-frequency oscillations used in this paper. These dynamics may be combined with time delays and methods such as the Balloon–Windkessel hemodynamic model (Friston et al., 2000) to model the BOLD signal (Cabral et al., 2011; Pope et al., 2023; Wu et al., 2022). Given our low-frequency range and the small size of the mouse brain, we did not include time delays or phase lags. Delays may be more relevant if we modeled fast oscillations (Cabral et al., 2011; Ghosh et al., 2008; Lee & Frangou, 2017).

Another attribute to consider when developing models for brain dynamics is structural connectivity architecture. The Kuramoto model has frequently been applied to undirected, MRI-based SCs, particularly from human subjects, though prior work has noted that simulations using undirected connectomes derived from directed data synchronized faster than those using the original directed connectomes (Crofts et al., 2022). Our comparative work showed a similar trend, as undirected connectomes more readily synchronized than directed connectomes. An analysis of our directed connectome architecture revealed that it was strongly directional, with individual edges showing a strong bias in connection direction between node pairs (Supporting Information Figure S8), perhaps explaining the significant difference in coupling dynamics between our two modeling approaches. Another study that applied the Kuramoto model to directed mouse connectivity data (Choi & Mihalas, 2019) found that its global synchronization behavior was powerfully dependent on strong, long-distance connections, so such connections may also have a greater effect on the undirected network. We also need to consider our simplification of using one connectome for all models, rather than an individual mouse connectome for each model. Prior research by Melozzi et al. (2019) found that FC predictions simulated using the reduced Wong Wang model on mouse MRI SCs could be improved using the SCs for individual animals rather than the average of these connectomes. However, the same study found that the tracer-based connectome significantly outperformed individual MRI data, suggesting that the detail added by using directed tracer information from the AMBCA connectome, rather than diffusion MRI estimation, improves predictions more than using individual data alone.

Although both our undirected and directed models were optimized to fit target data similarly well, the mean predictive power that we achieved without optimization was substantially lower than that of Melozzi et al. (2019; *R* = 0.488). While this may be due to the low complexity of the Kuramoto model, the recording conditions used to obtain empirical FC are another potential cause. Melozzi et al. recorded their fMRI data from a group of awake mice, while the fMRI dataset we used contained scans from multiple anesthetic conditions and demonstrated relatively low intersubject correlations. Differences between the populations of mice imaged with fMRI and injected for tracer data may also affect predictive power, as SC and FC characteristics can differ between distinct mouse populations (Karatas et al., 2021). The fMRI study we used (Grandjean et al., 2014) imaged female C57BL/6 mice, while the tracer-based connectome was determined from experiments in hundreds of male C57BL/6 J mice (Oh et al., 2014) and Melozzi et al. (2019) collected FC data from male B6/129P hybrid mice.

One broad goal for developing this model of mouse brain dynamics is to provide a predictive tool for how neural dynamics will change after specific brain lesions and to eventually correlate these changes in brain dynamics with cognitive function. Both structural connectivity and FC changes occur in humans after TBI (Caeyenberghs et al., 2017; Li et al., 2020; van der Horn et al., 2017a), and recent research suggests that both networks may predict symptoms after concussion (van der Horn et al., 2017b; Ware et al., 2023). Recent work that predicts injury using the features of brain networks after simulated impacts (Anderson et al., 2020; Wu et al., 2022) only examines the diagnosis—concussion or no concussion—and does not predict behavioral changes. Our approach to modeling injury was inspired by the neurodegeneration observed after an in vivo injury model (Chen et al., 2014) and aimed to represent the consequences of a mouse concussion model more closely than prior research using lesions to identify the most important brain areas regulating FC and dynamics (Alstott et al., 2009; Váša et al., 2015). However, our specific findings concerning changes in structural global efficiency, simulated FC, and their correlation should be confirmed for different approaches to injuring brain regions and different SC. Having applied our Kuramoto model to an experimental model of TBI, we could begin a more precise study of the relationship between lesion patterns in TBI and neurobehavioral outcomes.

A number of observations from our model are consistent with past studies: Simulated network dynamics and FC are disrupted by network injury (Alstott et al., 2009; Wu et al., 2022), a small group of nodes can have a significant influence on global efficiency (Anderson et al., 2020), and injury will produce areas of functional hypo- and hyperconnectivity (Bittencourt et al., 2022; Messé et al., 2013; Sharma et al., 2023; Zhou et al., 2012). Interestingly, we found that models optimized with directed connectivity were more consistently disrupted by simulated injury than those optimized with undirected connectivity, though this finding appears sensitive to changes in global coupling strength (Supporting Information Figure S6). This may be related to the higher sensitivity of synchrony on undirected networks to global coupling relative to directed networks, or to differences specific to the dynamics of the optimized simulations such as the broader, lower synchrony distribution and lower metastability distribution we found in the optimized, undirected simulations relative to the optimized, directed simulations (Supporting Information Figure S5). As neurodegeneration occurs over time after experimental TBI (Chen et al., 2014; Pleasant et al., 2011), future models should investigate connectivity loss in additional areas. Indeed, study of human subjects has suggested that structural global efficiency decreases that are not previously apparent can develop 1 year after concussion in adolescent patients (Chung et al., 2019). On the other hand, patients with concussion and reported PPCS complaints 3 months postinjury may present with higher structural global efficiency 4 weeks postinjury than those with concussion and no complaints (van der Horn et al., 2017a). Understanding longitudinal changes in mouse brain global efficiency and their relationship to behavioral impairments could provide additional insight into understanding how the human brain can change and recover after concussion.

The predicted emergence of hypoconnectivity and hyperconnectivity is intriguing, as it implies that neurodynamics can immediately compensate for physical loss in connectivity after TBI. However, the exact regions that we find to demonstrate relatively high likelihoods of hypoconnectivity and hyperconnectivity may be sensitive to the initial conditions and target FC network used to optimize each model, and the robustness of these exploratory findings under different simulation conditions and computational models should be investigated by future researchers. Furthermore, the mild CCI study from which we selected regions of interest to injure did not measure FC for a direct comparison. Our hypoconnectivity findings accord with recent calcium imaging experiments that found that cortical FC in mice was decreased after a CCI injury, particularly for interhemispheric connections and connections within the ipsilateral hemisphere, though these deficits slowly improved between 3 and 21 days postinjury (Bottom-Tanzer et al., 2024). Regional FC hypoconnectivity has been observed near a severe CCI lesion in rats 7 and 28 days postinjury using fMRI (Harris et al., 2016). This mirrors the predominant hypoconnectivity that we predicted in our model and is similar to the hypoconnectivity observed after mouse CCI, but this finding was weaker on day 14, and regional hyperconnectivity also emerged early and were most persistent for subcortical and contralateral cortical regions. Severe rat CCI has been shown to produce motor deficits in prior work (Harris et al., 2010), similar to the finding of early rotarod deficits caused by mild CCI in mice (Chen et al., 2014), which suggests that our hypoconnectivity predictions may be behaviorally relevant, as regions that were likely to display hypoconnectivity across our models included the primary motor area (Supporting Information Table S1). Hyperconnectivity during resting-state mouse fMRI has also been reported when measured 2 and 14 days postinjury for a closed-head concussion model (To & Nasrallah, 2021), though this finding was strongest for local changes in connectivity (regional homogeneity) and not identified on day 7, demonstrating the significance of time after injury to empirical findings. Additional rat research using EEG following fluid percussion injury has found postinjury hyperconnectivity to emerge early and change differently over time depending on the methods used to produce networks (Fox et al., 2024). Interestingly, increased FC between the cerebellum and amygdalar regions—similar to our model predictions of contralateral amygdala hyperconnectivity—was reported in another rat model after a single concussion (Kulkarni et al., 2019). As the original mouse mild CCI study could not identify a fear conditioning deficit (Chen et al., 2014), our modeling results suggest that such compensatory hyperconnectivity in the contralateral fear circuitry could be behaviorally relevant. Future functional imaging of brain regions involved in fear behavior (Wheeler et al., 2013) can explore this possibility, as injury severity, neurodegeneration changes at least 8 days after mild CCI (Chen et al., 2014), and longitudinal FC changes may influence both brain activity and behavior. Recent research has also predicted global decreases in simulated FC similar to empirical data when modeling mouse brain thalamic lesions or cortical inhibition by increasing the excitability of specific regions (Rabuffo et al., 2023). To study FC changes after injury with more realism and detail, future injury models should combine changes in regional activity with the connectivity loss we implemented in this work, which may also be achievable in future Kuramoto model studies by altering nodal oscillation frequencies (Wu et al., 2022).

Our modeling process demonstrates that directly representing the BOLD signal with the Kuramoto model can estimate FC. We further demonstrate that directed connectivity may improve the fidelity of FC predictions and change simulations more consistently in response to injuries when compared with undirected connectivity and compare our findings to rodent TBI literature. Given the prevalent use of undirected network measures, researchers should consider the importance of direction in brain connectomes when possible. For both architectures, we demonstrate that nodal frequency optimization can more closely fit models to individual FC networks, which may improve the accuracy of future studies that simulate FC or predict cognition from dynamical models. By investigating the effects of injury on mouse connectome simulations, our work proposes a means to interrogate theoretical research through experiments that compare predicted changes in brain dynamics to in vivo measurements. Researchers may model lesions or more complex injuries that incorporate finite element modeling (Chen et al., 2014; Pleasant et al., 2011) and neurodegeneration predictions (Kraft et al., 2012; Wu et al., 2022) to determine how these affect FC networks and behavior. Future studies combining computational and experimental research will compare their results to observational studies of TBI patients, which suggest that postinjury FC, whether measured or simulated, may predict functional deficits.

## ACKNOWLEDGMENTS

This work was funded by the Paul G. Allen Frontiers Group, grant 12347. The mouse brain SC we used was developed and published by Oh et al. (2014) for the AMBCA. Mouse BOLD fMRI data used in this research were made available by Radboud University and Dr. Joanes Grandjean.

## SUPPORTING INFORMATION

Supporting information for this article is available at https://doi.org/10.1162/netn_a_00431.

## AUTHOR CONTRIBUTIONS

Adam C. Rayfield: Conceptualization; Formal analysis; Investigation; Methodology; Software; Visualization; Writing – original draft; Writing – review & editing. Taotao Wu: Methodology; Software; Validation; Writing – review & editing. Jared A. Rifkin: Methodology; Validation; Writing – review & editing. David F. Meaney: Conceptualization; Funding acquisition; Supervision; Validation; Writing – review & editing.

## FUNDING INFORMATION

David F. Meaney, Paul G. Allen Frontiers Group (https://dx.doi.org/10.13039/100017023), Award ID: 12347.

## DATA AND CODE AVAILABILITY

The original mouse brain tract-tracing experiments were conducted for the AMBCA (https://connectivity.brain-map.org/); we used the directed 213-region connectome from supplementary data published by Oh et al. (2014) to produce the initial 426-region connectome and its reduced forms for our simulations and analysis. The fMRI data are available through the Radboud Data Repository (https://data.ru.nl/collections/di/dcmn/DSC_4180000.18_502). Code used for the optimization algorithm is available at the Meaney Lab website (https://www.seas.upenn.edu/~molneuro/software.html).

## Supplementary Material



## TECHNICAL TERMS

Kuramoto model: A mathematical model describing dynamics on a network of connected, oscillating elements.
Anterograde tracing: Labeling projections in the brain using an injection of a tracer, such as a fluorescent viral vector, at their source.
Strength: The sum of edge weights connected to a node on a weighted network.
Coefficient of variation: A measure of variability in data, calculated as the standard deviation divided by the mean.
Synchrony: Phase similarity among a group of oscillators.
Metastability: Variability in synchrony or states of synchronization over time among a group of oscillators.
Control energy: The integral of a node’s optimal control input, squared, over time.
Optimal control input: Approximate input required at a node to transition the functional network from an initial state to a target state.
Controlled cortical impact: An experimental method to model traumatic brain injury by impacting a model animal’s brain surface.
Global efficiency: The average inverse shortest path length between pairs of nodes in a network.
