# Supplementary material for: Individualized mouse brain network models produce asymmetric patterns of functional connectivity after simulated traumatic injury
Source: Netw Neurosci. 2025 Mar 20;9(1):326–51. doi: 10.1162/netn_a_00431 (PMC11949614; doi:10.1162/netn_a_00431)
Supplement: Supplementary file 1 [file netn-9-1-326-s001.pdf]

Supplementary Data

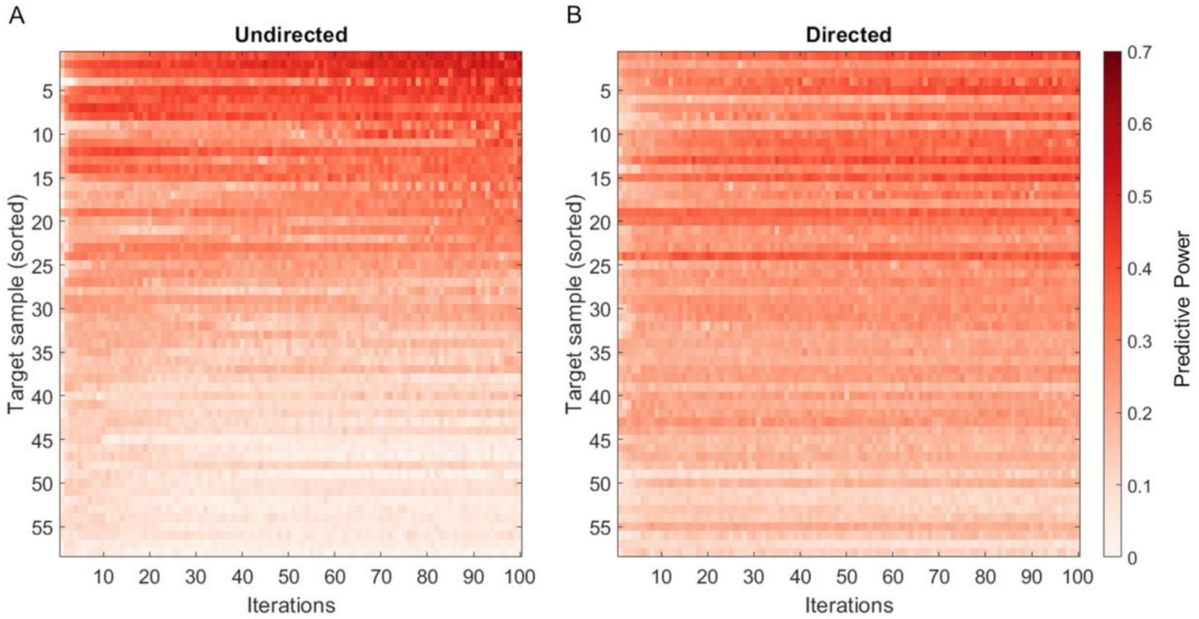

Figure S1: Predictive power of the target empirical FC during all 100 iterations of the optimization algorithm for simulations optimized to fit 58 empirical FCs on (A) the undirected network and (B) the directed network. For a given sample, the frequency distribution which maximized correlation with the target empirical data was selected to be the optimized model for that sample. Target samples in (A) and (B) are sorted by descending order of the maximum correlation achieved to empirical FC for the undirected model.

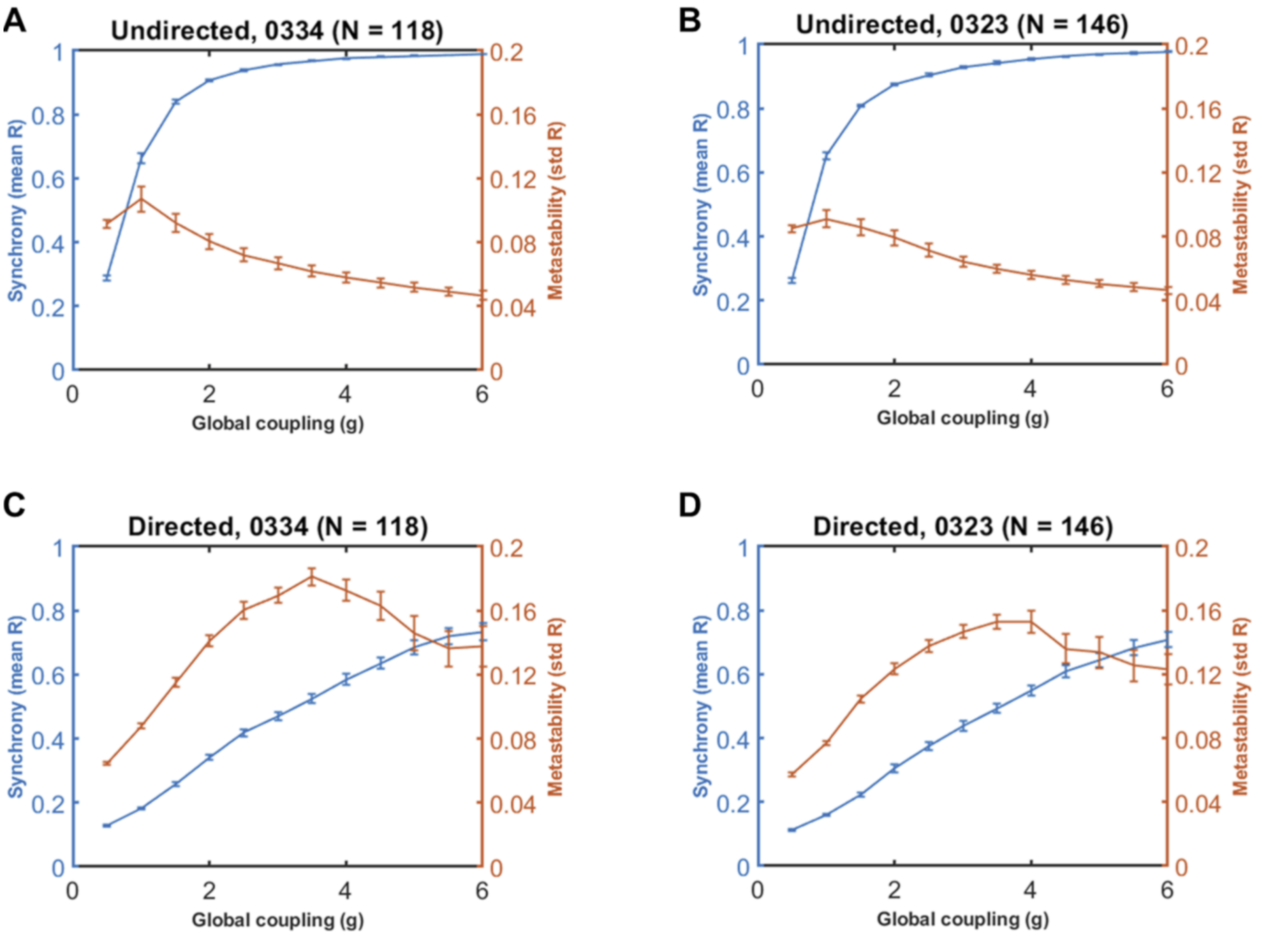

Figure S2: After reducing the number of ROIs to prepare for the optimization algorithm, dynamics were explored on the smallest (target sample 0334, N = 118) and largest (target sample 0323, N = 146) reduced networks to be similar to the trends found in the baseline undirected and directed networks. Synchrony and metastability vs. global coupling are shown for (A) the smallest undirected network, (B) the largest undirected network, (C) the largest directed network, and (D) the largest directed network. The coupling strengths of maximum metastability were used for the main analyses of the optimization algorithm (undirected network: g = 1.0, directed network: g = 3.5).

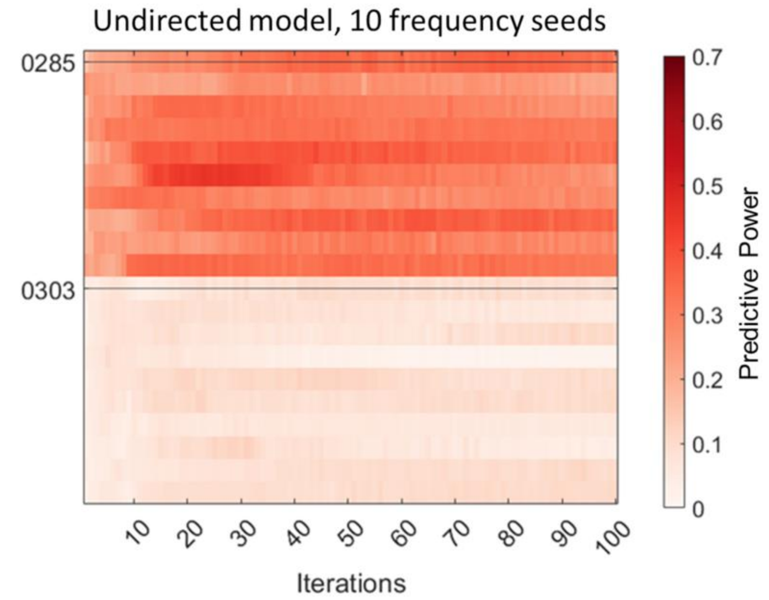

Figure S3: When using running the optimization algorithm using 10 different initial frequency distributions for 2 different target FCs (0285 from Iso1.5, 0303 from Med0.1), the predictive power depended more on the difference between the target samples than random variation due to the optimization algorithm's initial frequency seed (0285 mean predictive power = 0.37, 0303 mean predictive power = 0.11, $p < 0.0001$).

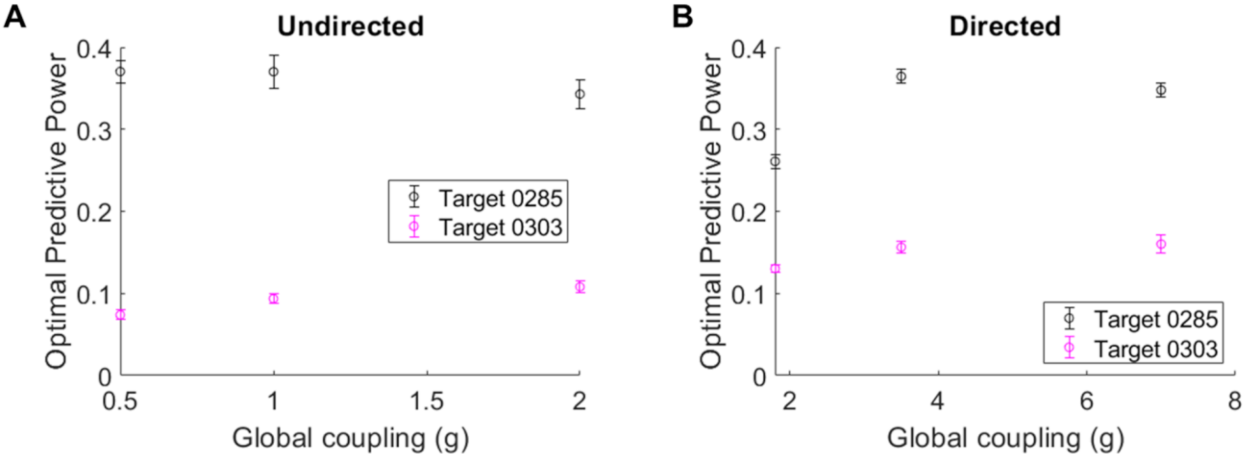

Figure S4: Optimal predictive power was significantly different between 2 different target FCs (0285 from Iso1.5, 0303 from Med0.1) when doubling or halving the approximate global coupling strength of maximum metastability. (A) On undirected structural connectomes, two-

way ANOVA confirmed that optimal predictive power was significantly affected by the target FC ($p < 0.0001$), but not by coupling strength or the interaction between target and coupling strength ($p > 0.05$). (B) On directed structural connectomes, two-way ANOVA confirmed that optimal predictive power was affected by the target FC ($p < 0.0001$), coupling strength ($p < 0.0001$), and their interaction ($p < 0.001$). Simulations were conducted using optimizations starting from the first 5 random frequency distributions used in Figure S3.

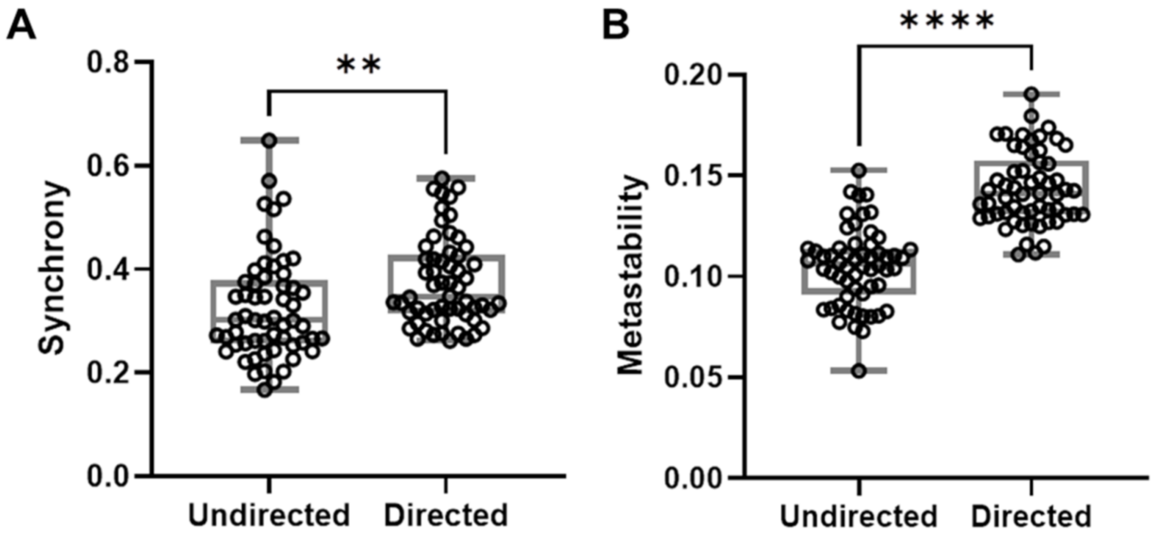

Figure S5: After optimization, both (A) synchrony and (B) metastability were significantly greater for the directed models than for the undirected models (**: $p < 0.01$, ****: $p < 0.0001$).

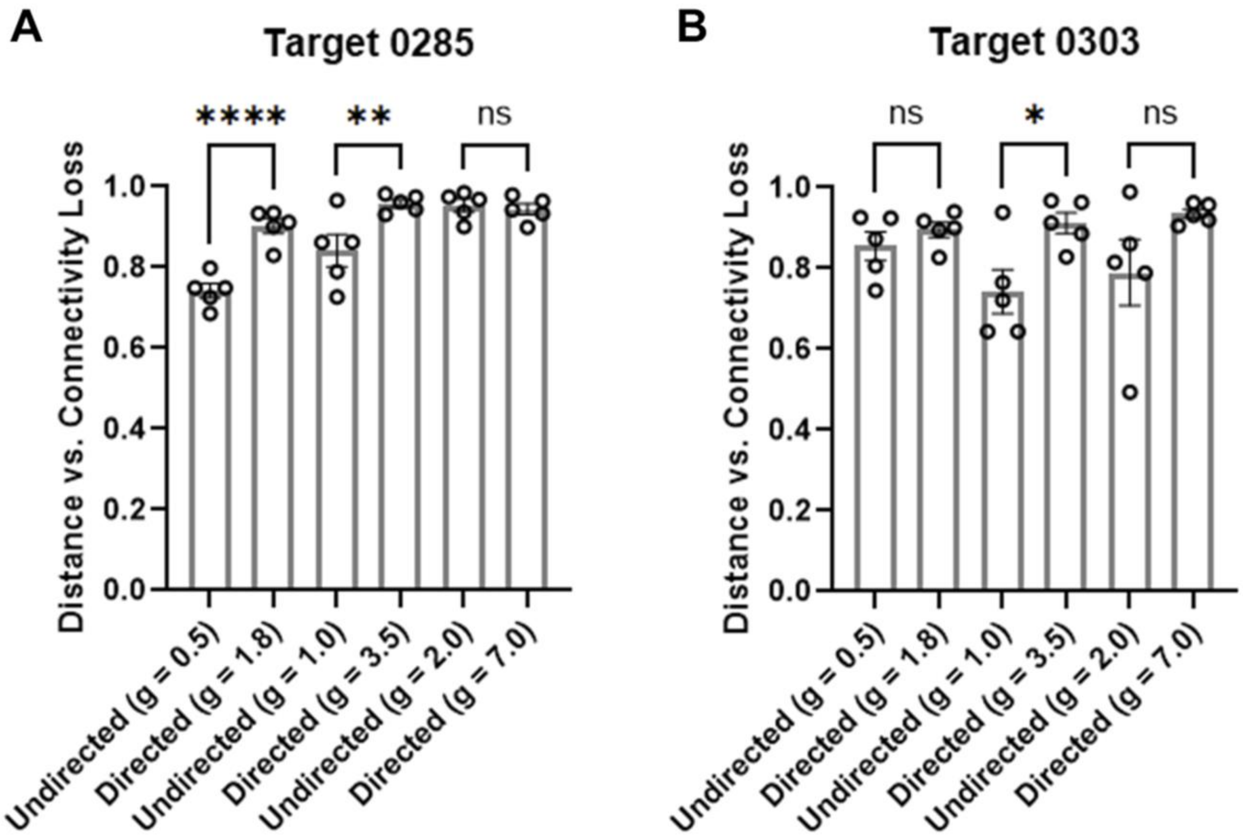

Figure S6: For the optimized models simulated at multiple coupling strengths in Figure S4, differences previously in the consistency of response to connectivity loss between the use of undirected and directed connectivity were affected by global coupling and target FC. (A) Differences between undirected and directed models optimized to fit FC 0285 were not statistically significant at the highest coupling strengths. (B) Differences between undirected and directed models optimized to fit FC 0303 were only significant at the coupling strength of maximum metastability. Five optimized models starting from different initial conditions were subjected to connectivity loss for each group, and multiple comparisons testing was conducted between specific groups of undirected and directed models. (ns: $p > 0.05$, *: $p < 0.05$, **: $p < 0.01$, ****: $p < 0.0001$). To confirm the source of these differences, two-way ANOVA was run for both undirected and directed models. The interaction between target sample and coupling

strength affected the consistency of undirected models’ response to injury ($p < 0.05$), while neither variable individually or in combination affected directed models.

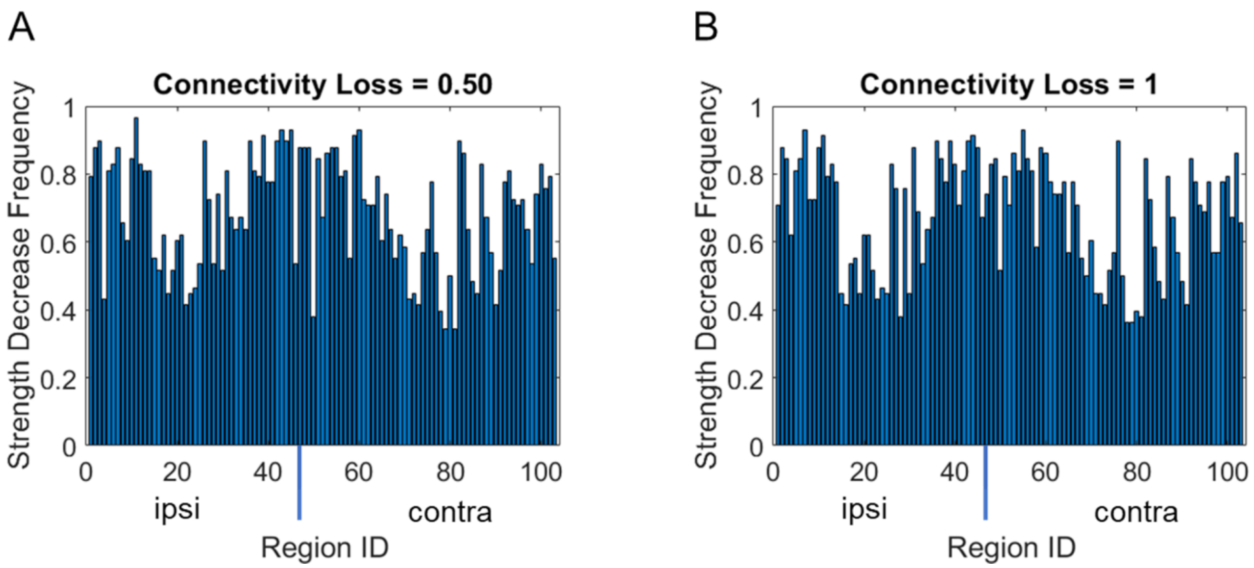

Figure S7: Frequency distributions of nodal strength decrease at levels of injury not shown in Figure 8. (A) The frequency distribution of strength decreases at connectivity loss = 0.50 was not significantly different from the distribution at connectivity loss = 0.25 (K-S $p = 0.11$, ns). (B) The frequency distribution of strength decreases at connectivity loss = 1 was not significantly different from the distribution at connectivity loss = 0.75 (K-S $p = 0.69$, ns).

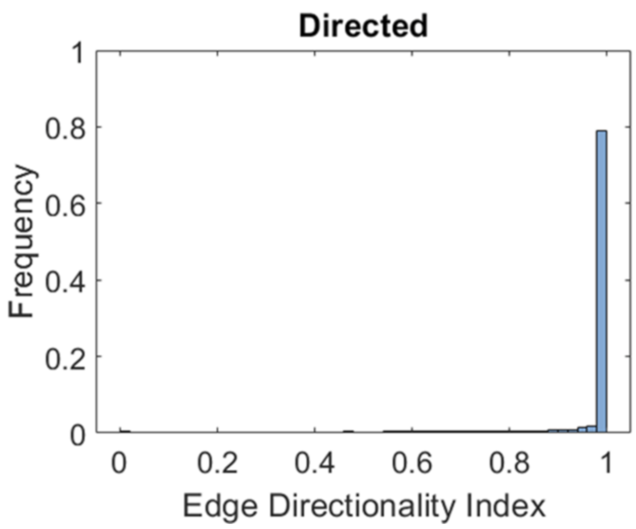

Figure S8: A histogram of “edge directionality index” in the directed mouse brain structural connectome. To quantify the relative directionality of edges throughout the directed mouse brain structural connectivity network that is lost by assuming undirected connectivity, we computed an “edge directionality index” between every connected pair of nodes in the directed network. For a pair of directed edge strengths $\{A_{ba}, A_{ba}\}$ between nodes a and b, this value was equal to $\frac{|A_{ba}-A_{ab}|}{A_{ba}+A_{ab}}$, which is 0 if the edge strengths in both directions are equal and 1 if there is only nonzero connectivity in one direction. The mean value on the directed network was 0.9209 (standard deviation 0.2042), demonstrating that a large proportion of edges in the directed network were not reciprocated by comparably strong edges in the opposite direction. This aspect of directed connectivity is completely lost by using an undirected connectome, for which the index is 0 for each pair of connected nodes.

**Brain regions of most frequent strength decrease**	**Strength decrease frequency**
Midbrain reticular nucleus, ipsilateral	0.9655 (56/58)
Primary somatosensory area, upper limb, ipsilateral	0.9483 (55/58)
Posterior complex of the thalamus, ipsilateral	0.9483 (55/58)
Primary motor area, ipsilateral	0.9310 (54/58)
Globus pallidus, external, ipsilateral	0.9310 (54/58)

Primary auditory area, contralateral	0.9310 (54/58)
Ventral auditory area, ipsilateral	0.9138 (53/58)
Caudoputamen, ipsilateral	0.9138 (53/58)
Lateral dorsal nucleus of the thalamus, ipsilateral	0.9138 (53/58)
Superior colliculus, motor related, ipsilateral	0.9138 (53/58)
Periaqueductal gray, ipsilateral	0.9138 (53/58)
**Brain regions of most frequent strength increase**	**Strength increase frequency**
Posterior amygdalar nucleus, contralateral	0.7241 (42/58)
Bed nuclei of the stria terminalis, contralateral	0.6379 (37/58)
Primary somatosensory area, nose, contralateral	0.6207 (36/58)
Postpiriform transition area, contralateral	0.6207 (36/58)
Lateral amygdalar nucleus, contralateral	0.6207 (36/58)
Medial amygdalar nucleus, contralateral	0.5862 (34/58)
Taenia tecta, ipsilateral	0.5690 (33/58)

Olfactory tubercle, ipsilateral	0.5690 (33/58)
Cortical amygdalar area, posterior, contralateral	0.5690 (33/58)
Endopiriform nucleus, ventral, contralateral	0.5690 (33/58)
Basomedial amygdalar nucleus, contralateral	0.5690 (33/58)

Table S1: List of analyzed nodes (i.e. 103 regions common to all 58 optimized models and not directly injured) which most frequently decreased in strength and which most frequently increased in strength among our models after a specific injury level (connectivity loss = 0.75), and their corresponding frequency of decreased or increased strength among the optimized models. Regions visualized in Figure 8C.
